# Predictors of diagnostic yield in bronchoscopy: a retrospective cohort study comparing different combinations of sampling techniques

**DOI:** 10.1186/1471-2466-8-2

**Published:** 2008-01-26

**Authors:** Kjetil Roth, Jon A Hardie, Alf H Andreassen, Friedemann Leh, Tomas ML Eagan

**Affiliations:** 1 Dept. of Thoracic Medicine, Haukeland University Hospital, Bergen, Norway; 2 Dept. of Internal Medicine, Aalesund Hospital, Aalesund, Norway; 3 Dept. of Pathology, Haukeland University Hospital, Bergen, Norway

## Abstract

**Background:**

The reported diagnostic yield from bronchoscopies in patients with lung cancer varies greatly. The optimal combination of sampling techniques has not been finally established.

The objectives of this study were to find the predictors of diagnostic yield in bronchoscopy and to evaluate different combinations of sampling techniques.

**Methods:**

All bronchoscopies performed on suspicion of lung malignancy in 2003 and 2004 were reviewed, and 363 patients with proven malignant lung disease were included in the study. Sampling techniques performed were biopsy, transbronchial needle aspiration (TBNA), brushing, small volume lavage (SVL), and aspiration of fluid from the entire procedure. Logistic regression analyses were adjusted for sex, age, endobronchial visibility, localization (lobe), distance from carina, and tumor size.

**Results:**

The adjusted odds ratios (OR) with 95% confidence intervals (CI) for a positive diagnostic yield through all procedures were 17.0 (8.5–34.0) for endobronchial lesions, and 2.6 (1.3–5.2) for constriction/compression, compared to non-visible lesions; 3.8 (1.3–10.7) for lesions > 4 cm, 6.7 (2.1–21.8) for lesions 3–4 cm, and 2.5 (0.8–7.9) for lesions 2–3 cm compared with lesions <= 2 cm. The combined diagnostic yield of biopsy and TBNA was 83.7% for endobronchial lesions and 54.2% for the combined group without visible lesions. This was superior to either technique alone, whereas additional brushing, SVL, and aspiration did not significantly increase the diagnostic yield.

**Conclusion:**

In patients with malignant lung disease, visible lesions and larger tumor size were significant predictors of higher diagnostic yield, after adjustment for sex, age, distance from carina, side and lobe. The combined diagnostic yield of biopsy and TBNA was significant higher than with either technique alone.

## Background

The incidence of lung cancer in Norway increased from 21.1/100 000 person years in 1967–1971 to 36.1/100 000 in 2000–2004 for men and from 4.5/100 000 to 21.1/100 000 for women [1]. Bronchoscopy is the main diagnostic procedure in patients with endoscopic visible lesions. British Thoracic Society (BTS) guidelines from 2001 recommend biopsies, brushings, and washings for sampling from visible lesions. The diagnostic yield for visible lesions should be at least 80% [2]. Transbronchial needle aspiration (TBNA) was not included in the recommendation, and the optimal combination of sampling techniques in peripheral lesions was not settled [2].

Computer tomography (CT) guided sampling techniques have a diagnostic yield of approximately 90% in peripheral lung cancer [3], with the disadvantage of a high incidence of pneumothorax [4]. Previous studies of bronchoscopy in peripheral lesions have shown a great variability in the diagnostic yield, with sensitivity for cancer between 20% and 86% [5-9]. The reported predictors of positive samples have been size [6,9-16], location [10,13,14,17], visible lesion, compression or constriction [12,18,19], CT bronchus sign [13,17,20], fuzzy or sharp border [11], the use of a C-arm fluoroscope [8,21], and sampling technique [11,14,15,18,22-29]. Many studies were based on bronchoscopies performed by selected investigators in highly specialized centres [5,8,10,18].

The great variability in the previous studies makes it difficult to know if the real life situation in a clinical practice is comparable with the reported results. The choice of sampling techniques is often left to the physician who performs the bronchoscopy, and it is not known if a standardised approach gives better results.

The aims of this study were to evaluate the sensitivity of bronchoscopy for detecting malignant disease in clinical practice, identify predictors of a high diagnostic yield, and to evaluate different pairs of sampling techniques.

## Methods

All 1438 bronchoscopies performed between January 2003 and December 2004 at Haukeland University Hospital, Bergen, Norway, were retrospectively reviewed. All procedures where the indication for bronchoscopy was to obtain samples from a lesion suspicious of malignancy and where the final diagnosis obtained through all possible methods was malignant lung disease were eligible for inclusion in the study. If a patient had repeated bronchoscopies, only the first bronchoscopy was included.

Of 493 patients with a lesion suspicious of malignancy, 367 patients were investigated with bronchoscopy of later proven malignant disease. Three patients were excluded because no samplings had been performed during bronchoscopy, and one patient was excluded because it was not possible to perform the bronchoscopy. Thus, 363 patients were included in the study sample.

Twenty three medical doctors; nine pulmonologists and fourteen trainees (pulmonary residents and fellows) performed the bronchoscopies. The investigations were performed with Olympus BF 1T 160 bronchoscopes, using Boston "Radial Jaw3" for biopsies, Boston 21 Gauche "Stifcor" transbronchial aspiration needle for TBNA, and Boston "Cellebrity" for brushings. TBNA was taken directly from endobronchial lesions, under visual control from constriction and compression, and blind or under fluoroscopic guidance from peripheral lesions. The procedures were performed transorally without an endotracheal tube. Patients were semi-sedated with pethidine hydrochloride 25–75 mg or midazolam 2.5–5 mg. Biopsies and small volume lavage (SVL), a bronchial washing with 10–20 ml saline were fixated in formalin. TBNA and brushings were alcohol fixed on a glass slide. In addition, aspiration from the whole bronchoscopy was collected and a sample of 10–20 ml was fixated in formalin.

Two of the authors (KR and TME) registered endobronchial visibility and indications for bronchoscopy based on a review of the bronchoscopy reports. Endobronchial visibility was categorized into 1) *visible lesion*, 2) *constriction, compression or suspected submucosal changes*, or 3) *non-visible lesion*. The largest size of the lesion was measured from the CT scan in all but five cases, in which size was estimated from the chest radiograph. The distance from carina to the lesion was measured on a posterior-anterior chest radiograph or on a reconstruction from the CT scan. In 40 cases the distance from carina was impossible to measure. The localization (side and lobe) of the lesion was registered from the CT scan. In cases of multiple lesions, the sampled lesions were registered. The cases were categorized as indeterminate when it was impossible to decide which lesion that had been sampled.

Malignant lung disease was defined as positive histological or cytological results or certain malignant disease after clinical follow up. The Department of Pathology provided a computer-based search in the registry of systemized nomenclature of medicine (SNOMED) codes from all bronchoscopies, ultrasound guided transthoracic needle aspirations, CT guided samplings, and operations. Cells suspicious of malignancy usually lead to further investigations and were not included in the definition of positive diagnostic yield. The diagnostic yield in this study was defined as sensitivity for cancer. The gold standard was defined as histological proven malignant disease or clinical malignant disease during follow up. Three sources of information were used to avoid exclusion of patients with later proven malignant disease: 1) A computer based search through patient journals for a later malignant diagnosis. 2) A review of the journals of all patients who died before November 2005. 3) Follow up until November 2005 of all patients discharged with a diagnosis of an uncertain pulmonary lesion.

The statistical analyses were performed in SPSS, using Chi square tests for univariate analyses, multivariate logistic regression to estimate the odds ratios and adjust for confounding, and McNemar's test to compare different sampling techniques. The Regional Norwegian Ethical Committee and the Norwegian Social Science Data Service approved the study.

## Results

The baseline characteristics of the patients are displayed in Table 1. The first bronchoscopy provided a conclusive diagnosis of malignant disease in 161 of the 363 patients (44%). Two patients had cytological specimens suspicious of cancer in the first bronchoscopy, with no other sampling techniques to confirm the diagnosis. Almost 40% of the patients diagnosed with cancer these two years were women.

**Table 1:** Baseline characteristics of 363 cases

	n	%
***Sex***		
Male	221	60.9
Female	142	39.1
***Age (years)***		
< 59	87	24.0
59–67	92	25.3
68–74	90	24.8
> 74	94	25.9
Size*		
<= 2 cm	41	11.3
2–3 cm	62	17.1
3–4 cm	53	14.6
> 4 cm	207	57.0
***Distance from carina#***		
<= 5 cm	144	39.7
> 5 cm	179	49.3
Indeterminate	40	11.0
***Endobronchial visibility***		
Non visible lesion	132	36.4
Constriction, compression or submucosal lesion	90	24.8
Visible lesion	141	38.8
***Localization***		
Left upper lobe without lingula	75	20.7
Left lingula	10	2.8
Left lower lobe	53	14.6
Right upper lobe	88	24.2
Right middle lobe	19	5.2
Right lower lobe	73	20.1
Mediastinum	30	8.3
Indeterminate	15	4.1

*Size was measured on axial Ct thorax, only chest radiograph available in 5 cases.

#Distance from carina is measured as the distance from carina to the lesion on the chest radiograph, front projection.

The final diagnostic method and pathological classification is shown in Table 2. Transthoracic sampling techniques provided the diagnosis in 105/363 patients. Of the transthoracic samples, 87 were obtained by CT guided sampling, 12 by ultrasound guided sampling, two were pleural biopsies, and four were pleural effusions (Table 2).

**Table 2:** The diagnostic method and final diagnosis of 363 cases with malignant lung disease.

	n	%
***Final diagnostic method***		
First bronchoscopy*	163	44.9
Repeated bronchoscopy	28	7.7
Transthoracic sampling	105	28.9
Mediastinoscopy	2	0.6
Operation, autopsy and open lung biopsy	11	3.0
Sampling from other organs than the lung	15	4.1
Clinical diagnosis of cancer	17	4.7
Malignant diagnosis obtained before bronchoscopy	22	6.1
***Pathology***		
Small cell lung cancer	53	14.6
Adenocarcinoma	100	27.5
Squamous cell carcinoma	66	18.2
Large cell carcinoma	10	2.8
Non classifiable non small cell lung cancer.	82	22.6
Metastasis to the lung	21	5.8
Other cancer in the lung#	14	3.9
Only clinical diagnosis	17	4.7

* 2 patients diagnosed with uncertain pathology.

#Other cancer in the lung: Carcinoid tumor:6, Lymphoma:5, Mesothelioma:2,

Neuroendocrine tumor:1.

Table 3 presents the diagnostic yield of the different bronchoscopic sampling techniques. The sampling techniques performed were biopsy (201/363), TBNA (191/363), brushing (187/363), SVL (72/363), and aspiration of fluid from the entire procedure (356/363). Biopsy consistently gave the highest diagnostic yield with the possible exception in the case of lesions smaller than 2 cm. In univariate analyses endobronchial visibility, tumor size, and distance from carina were predictors of a higher diagnostic yield (Table 3). The overall sensitivity for cancer increased from 16.7% in non-visible lesions, to 34.4% for compression, constriction or submucosal disease, and further to 76.6% in visible lesions (χ^2^: p < 0.001).

**Table 3:** Diagnostic yield of different sampling techniques

	Biopsy (n = 201)	TBNA (n = 191)	Brushing (n = 187)	SVL (n = 72)	Aspiration (n = 356)	All (n = 363)
**Overall diagnostic yield**	122/201 (60.7)	78/191 (40.8)	43/187 (23.0)	5/72 (6.9)	29/356 (8.1)	161/363 (44.4)
						
**Endobronchial visibility**	**	**	**		**	**
Non visible lesion	9/36 (25.0)	4/21 (19.0)	13/68 (16.0)	3/46 (6.5)	5/130 (3.8)	22/132 (16.7)
Constriction/compression	22/48 (45.8)	17/69 (24.6)	5/49 (10.2)	1/11 (9.1)	2/88 (2.3)	31/90 (34.4)
Visible lesion	91/117 (77.8)	57/101 (56.4)	25/57 (43.9)	1/15 (6.7)	22/138 (15.9)	108/141 (76.6)
						
**Tumor size**	*					**
<= 2 cm	3/13 (23.1)	4/16 (25.0)	2/19 (10.5)	0/13 (0.0)	2/41 (4.9)	7/41 (17.1)
> 2 cm and <= 3 cm	15/24 (62.5)	6/18 (25.0)	4/35 (11.4)	0/19 (0.0)	6/59 (10.2)	20/62 (32.3)
> 3 cm and <= 4 cm	19/35 (54.3)	12/24 (50.0)	8/31 (25.8)	1/13 (7.7)	4/51 (7.8)	25/53 (47.2)
> 4 cm	85/129 (65.9)	56/127 (44.1)	29/102 (28.4)	4/27 (14.8)	17/205 (8.3)	109/207 (52.7)
						
**Distance to carina**	*		*			*
<= 5 cm	59/86 (68.6)	40/93 (43.0)	23/68 (33.8)	0/12 (0.0)	13/142 (9.2)	78/144 (54.2)
> 5 cm	44/88 (50.0)	26/74 (35.1)	17/102 (16.7)	3/52 (5.8)	12/165 (6.8)	62/179 (34.6)
Indeterminate	19/27 (70.4)	12/24 (50.0)	3/17 (17.6)	2/8 (25.0)	4/37 (10.8)	21/40 (52.5)
						
**Side**						
Left	49/80 (61.3)	26/75 (34.7)	20/80 (25.0)	4/27 (14.8)	14/147 (9.5)	62/151 (41.1)
Right	68/115 (59.1)	46/106 (43.4)	22/103 (21.4)	1/42 (2.4)	14/195 (7.2)	92/198 (46.5)
Indeterminate	5/6 (83.3)	6/10 (60.0)	1/4 (25.0)	0/3 (0.0)	1/14 (7.1)	7/14 (50.0)
						
**Lobe**						
Upper lobe without lingula	47/92 (51.1)	29/91 (31.9)	20/92 (21.7)	3/27 (11.1)	15/162 (9.3)	68/164 (41.5)
Middle lobe/lingula	8/13 (61.5)	6/14 (42.9)	6/18 (33.3)	0/9 (0.0)	0/27 (0.0)	11/29 (37.9)
Lower lobe	50/74 (67.6)	29/58 (50.0)	13/60 (21.7)	2/30 (6.7)	11/124 (8.9)	61/127 (48.0)
Mediastinum	8/11 (72.7)	7/18 (38.9)	2/13 (15.4)	0/5 (0.0)	0/30 (0.0)	12/30 (40.0)
Indeterminate	9/11 (81.8)	7/10 (70)	2/4 (50.0)	0/1 (0.0)	3/13 (23.1)	9/13 (69.2)

*p < 0.05. **p < 0.001

Data are presented as number of positive samples/all samples (%). Statistical analysis: χ^2^.

TBNA = Transbronchial needle aspiration. SVL = Small volume lavage.

In non visible lesions the diagnostic yield using a C-arm fluoroscope was 17/48 (35.4%) compared to 4/83 (4.8%) without a C-arm fluoroscope (χ^2^: p < 0.001), data not shown in Table 3.

Table 4 presents predictors of diagnostic yield for each sampling technique in a multivariate analysis after adjustment for age, sex, lobe, endobronchial visibility, size and distance from carina. The adjusted odds ratio (OR) for a positive diagnostic result, was 2.6 (1.3–5.2) for constriction, compression or suspected submucosal changes and 17.0 (8.5–34.0) for visible lesions, compared to non visible lesions. Endobronchial visibility was a significant predictor for a higher diagnostic yield in all sampling techniques. Larger tumor size predicted an overall higher diagnostic yield, but was statistically significant only for biopsies. In the multivariate analyses, distance from carina and localization of the lesion did not predict the diagnostic yield.

**Table 4:** Predictors of a higher diagnostic yield. Odds ratio (95%CI) in multivariate analysis.

	Biopsy (n = 201)	TBNA (n = 191)	Brushing (n = 187)	Aspiration (n = 356)	All (n = 363)
***Endobronchial visibility***	**	**	*	**	**
Non-visible lesion	1	1	1	1	1
Compression/constriction	2.4 (0.8–7.0)	1.2 (0.3–4.4)	0.4 (0.1–1.5)	0.8 (0.1–4.6)	2.6 (1.3–5.2)
Visible lesion	10.8 (3.8–30.7)	5.0 (1.4–17.5)	3.1 (1.1–8.3)	6.4 (1.9–20.9)	17.0 (8.5–34.0)
***Tumor size***	*				*
<= 2 cm	1	1	1	1	1
2–3 cm	7.9 (1.4–45.1)	0.8 (0.2–4.0)	1.1 (0.2–7.3)	2.2 (0.4–12.7)	2.5 (0.8–7.9)
3–4 cm	9.4 (1.7–52.2)	3.1 (0.6–15.3)	2.8 (0.5–17.0)	2.1 (0.3–13.6)	6.7 (2.1–21.8)
> 4 cm	6.7 (1.5–30.6)	2.0 (0.5–7.6)	2.0 (0.4–10.9)	1.0 (0.2–5.4)	3.8 (1.3–10.7)

Odds ratio adjusted for age, sex, lobe and distance from carina. TBNA = Transbronchial needle aspiration.

* Likelihood ratio: p < 0.05. ** Likelihood ratio: p < 0.001.

Different pairs of sampling techniques were compared (Table 5). The sample size was not large enough to compare endobronchial visibility in three categories. Therefore non-visible lesions, compression, constriction, or suspected submucosal changes were combined to one category. In 86 patients with visible lesions, biopsy and TBNA were performed with a combined diagnostic yield of 83.7% (72/86, Table 5). In 38 of the 86 procedures, brushing was performed in addition to biopsy and TBNA, and in 85 of the procedures aspirations were also examined. Cytological examination of the brushings and aspirations provided an increased diagnostic yield of one case (NS). Compared to the combination of biopsy and TBNA in these 86 procedures, seven cases would have been missed without TBNA (p = 0.02), and 22 cases would have been missed without biopsy (p < 0.001). The result in the group with non-visible lesions, compression, constriction or suspected submucosal changes was similar. In this group, biopsy and TBNA was performed in 48 patients with a combined diagnostic yield of 54.2% (Table 5). In these procedures additional 47 aspirations and 25 brushings were performed, which increased the diagnostic yield by only one case (NS). The diagnostic yield with a combination of biopsy and TBNA was significantly higher than with biopsy or TBNA alone.

**Table 5:** Diagnostic yield of different combinations of sampling techniques

	The result of the first two sampling techniques in the combination	The result of all sampling techniques performed		The result of the first sampling technique (1)		The result of the second sampling technique (2)	
	DY (%)	DY (%)	p	DY (%)	p	DY (%)	p

**Non-visible lesion/compression or constriction:**							
Biopsy(1) and TBNA(2)	26/48 (54.2)	27/48 (56.3)	NS	20/48 (41.7)	0.03	16/48 (33.3)	0.002
Biopsy(1) and Brushing(2)	18/42 (42.9)	20/42 (47.6)	NS	14/42 (33.3)	NS	9/42 (21.4)	0.004
TBNA(1) and Brushing(2)	16/51 (31.4)	21/51 (41.2)	0.06	11/51 (21.6)	0.06	10/51 (19.6)	0.03
**Endobronchial visible lesion:**							
Biopsy(1) and TBNA(2)	72/86 (83.7)	73/86 (84.9)	NS	65/86 (75.6)	0.02	50/86 (58.1)	< 0.001
Biopsy(1) and Brushing(2)	37/46 (80.4)	39/46 (84.8)	NS	34/46 (73.9)	NS	23/46 (50.0)	< 0.001
TBNA(1) and Brushing(2)	30/47 (63.8)	39/47 (83.0)	0.004	27/47 (57.4)	NS	22/47 (46.8)	0.008

Data are presented as number of positive samples/all samples (%). Statistical analysis: McNemar's test. DY = Diagnostic Yield. All results are compared with the diagnostic yield of the respective combination of the two sampling techniques.

## Discussion

Endobronchial visibility and tumor size were the predictors of diagnostic yield in bronchoscopies of patients with malignant disease in this study. The diagnostic yield was 16.7% in procedures with non-visible lesions, 34.4% in procedures with compression, constriction or suspected submucosal changes, and 76.6% in procedures with endobronchial visible lesions. The combination of biopsy and TBNA had the highest diagnostic yield both in visible lesions and in the combined group of non-visible lesions, constriction, compression, or suspected submucosal changes. Biopsy and TBNA together was significant better than either technique alone.

There are some methodological issues to consider.

The main strength of this study is that it reflects the diagnostic value of bronchoscopy in a regular clinical practice. For many operative procedures, an important factor is the skill of the operator. The current study did not have the power to examine diagnostic yield by operator. However, the large number of operators partly reflects the large number of patients seen with lung cancer, at our centre. The results of this study should be comparable to centres that include trainees and where each doctor performs approximately thirty bronchoscopies per year.

Patient selection bias is a problem in studies of diagnostic yield in bronchoscopy. Haukeland University Hospital is the only centre for diagnosing lung cancer in Hordaland County and some smaller surrounding municipalities. Thus all patients from surrounding area would be included. The follow up time to December 2005 with inclusion of clinical cancer were important factors to ensure inclusion of all cancers present among those examined in 2003/04.

Another selection bias pertains to the choice of sampling methods. In our centre, the choice of sampling method is left to the judgement of the examiner. Thus, although in most bronchoscopies more than one sampling method was employed, for example biopsy and TBNA, rarely all five available techniques were used. In many procedures there is an urge to use the least amount of material and time necessary, in order to avoid complications and discomfort for the patients. Thus, an operator who feels to have obtained an adequate sample may terminate the procedure without all techniques being employed. On the other hand, some sampling techniques may be dropped if complications arise, or the tumor seems impossible to reach. Clearly this is a weakness in the current study, when comparing the different sampling methods. However, the current study reflects how bronchoscopies are normally performed. In studies where all possible techniques are used, there may be a selection bias in that patients where the procedure is terminated prior to full sampling, for instance due to complications, are excluded from the study. This bias would likely inflate the results from studies in which only patients having undergone all procedures are included.

Previous studies have categorized "compression, constriction or suspected submucosal changes" either with visible lesions or with non-visible lesions. Some studies have included these as non specific findings [12,30], thus increasing the diagnostic yield of peripheral lesions. Other studies have classified the findings as submucosal-peribronchial disease [31,32], decreasing the diagnostic yield of visible lesions. We have classified this group as a separate category. The finding that these lesions show a diagnostic yield intermediate between visible and non-visible supports our choice of classification. However, for the analyses in table 5, sample size dictated that non-visible lesions be grouped with "compression, constriction or suspected submucosal changes". As TBNA and biopsy were more often used for the later category, and brushings more often for non-visible lesions, there could be a tendency for the results of TBNA to appear better in the combined category.

The overall diagnostic yield of 77% in visible lesions, is similar to previously reported results [7,21,25,30,31]. The overall diagnostic yield of 17% in non visible lesions is lower than in some studies [3,5-15], but higher than the yield reported in a Scottish multi-centre study (9%) [30].

The individual diagnostic yield of biopsy, brushing or TBNA from visible lesions were similar to previous reported studies [18,21,28,29]. SVL and aspiration had lower diagnostic yields for visible and non visible lesions, compared with other studies [21,25,33]. Studies with bronchoalveolar lavage have had a higher diagnostic yield, possibly due to higher fluid volume [22,23]. The low diagnostic yield might also be explained by the procedure of taking a small sample of 10–20 ml from the fluid aspirated and the lack of wedging the bronchoscope into the affected bronchus. Although there are relatively few prospective trials, they tend to report a higher diagnostic yield than retrospective trials, which may be due to the benefits of planning [20,28]. Studies from highly specialized centres or from selected procedures with all sampling techniques, have a higher diagnostic yield compared to the current study [5,8,13].

The C-arm fluoroscope increased the diagnostic yield from 5% to 35%. The rate of fluoroscopy in the current study was low, mainly because the C-arm fluoroscope was operated by radiographers who were not always available. The current study was not powered to examine differences in diagnostic yield between procedures with and without the C-arm fluoroscope. However, this study suggests a significant benefit from using the fluoroscope.

Several studies have examined predictors for a higher diagnostic yield, but only in univariate analysis [9-11,13-16,18,20]. Significant predictors found have been size [10,14-16,20], location [9-11], and endobronchial visibility [18]. In the current study, endobronchial visibility and tumor size prevailed as significant predictors when several potential predictors were examined together. Stratified on each sampling technique, the effect of size was significant only for biopsy. Both brushing and TBNA gave better results in larger lesions, but the results were not statistically significant. This could indicate that sample size was too small to show the effect of size on these sampling methods. However, brushings might be sampled from a wider area than biopsy, and the size of the lesion might be less important for this method.

Knowing the diagnostic yield of each sampling technique is important for choosing the optimal combination [11,32-34]. In the current study biopsy was the most important sampling technique in patients with and without visible lesions. Addition of TBNA or brushing increased the diagnostic yield of biopsy, but three sampling techniques were not significantly better than two. Biopsy and TBNA had the highest combined diagnostic yield but the results must be interpreted cautiously. The most important reasons that the result could be biased was the retrospective nature of the study, because non visible lesions had to be categorized together with compression and constriction, and because of the low number of biopsy, brushing and TBNA. Although brushings did not prove significant additional benefit, too few procedures included brushings for the matter to be settled. However, the value of SVL and aspiration in addition to the other techniques seemed too small to warrant the effort. While BTS guidelines presently recommend a combination of biopsy, brushing, and washing, these data suggest that the combination of biopsy and TBNA may be superior for visible and non-visible lesions.

## Conclusion

This study evaluated predictors of diagnostic yield of bronchoscopy reflecting clinical real life. Endobronchial visible lesion and larger tumor size predicted a higher diagnostic yield. The diagnostic yield was comparable with previous studies for visible lesions but was lower than in many of the previous studies for non-visible lesions.

BTS guideline recommended biopsy, brushing and washing for visible lesions. This study has shown that biopsy and TBNA might be better. In the combined group of compression, constriction, suspected submucosal disease and non visible lesions, biopsy and TBNA was better than each sampling technique alone, however this study did not have sufficient power to determine whether brushing should be performed or not. These groups should be further investigated to find the optimal combination of sampling techniques and a cost effectiveness analysis could be performed. Washings were performed in almost all procedures and did not increase the diagnostic yield significantly in any groups.

## Abbreviations

BTS = British Thoracic Society, CI = confidence interval, CT = computer tomography, OR = odds ratio, SD = standard deviation, SNOMED = systemized nomenclature of medicine, SVL = small volume lavage, TBNA = transbronchial needle aspiration.

## Competing interests

The author(s) declare that they have no competing interests.

## Authors' contributions

TME and KR read the bronchoscopy reports and registered the information provided, viewed the chest radiographs and the CT scans, implemented the information from the pathological department, performed the statistical analysis, and wrote the manuscript.

JH and AHA participated in the design of the study, and reviewed the manuscript thoroughly.

FL provided all the information from the pathological department, was part of the planning of the study, and reviewed the manuscript thoroughly.

## Pre-publication history

The pre-publication history for this paper can be accessed here:


